# Longitudinal Characterization of Males With X-Linked Creatine Transporter Deficiency: Final Results of a Multiyear Observational Study

**DOI:** 10.1016/j.pediatrneurol.2025.10.023

**Published:** 2025-10-30

**Authors:** Judith S. Miller, Cristan Farmer, Susan Blair, Simona Bianconi, Natacha Akshoomoff, Irina Anselm, Bruce A. Barshop, Lindsey Becker, Amanda E. Bennett, Leandra N. Berry, Elizabeth M. Berry-Kravis, Aleksandra Bruchey, Anna W. Byars, Tricia Cimms, Kim M. Cecil, Maxine Covello, Laura S. Cubit, Tanvi Das, Robert J. Davis, Madison Drye, Can Ficicioglu, John B. Fulton, Robin P. Goin-Kochel, Whitney Guthrie, Barbara E. Hallinan, Fady Hannah-Shmouni, Kathryn E. Gustafson, Dwight D. Koeberl, Nicola Longo, Eva Mamak, Saadet Mercimek-Andrews, Claire Michalak, Forbes D. Porter, Samar Rahhal, Linda Rees, Gail A. Spiridigliozzi, Caitlin Stone, Nancy R. Sullivan, V. Reid Sutton, Rebecca P. Thomas, Manisha Udhnani, Susan Waisbren, Michelle Xu, Lin Zhang, Melanie Brandabur, Audrey Thurm

**Affiliations:** a Center for Autism Research, Children’s Hospital of Philadelphia, Philadelphia, Pennsylvania; b Perelman School of Medicine, University of Pennsylvania, Philadelphia, Pennsylvania; c Neurodevelopmental and Behavioral Phenotyping Service, Office of the Clinical Director, National Institute of Mental Health, Bethesda, Maryland; d Ultragenyx Pharmaceutical Inc., Novato, California; e Formerly Division of Translational Medicine, Eunice Kennedy Shriver National Institute of Child Health and Human Development, National Institutes of Health, Bethesda, Maryland; f Department of Pediatrics, University of California San Diego, La Jolla, California; g Department of Neurology, Boston Children’s Hospital, Harvard Medical School, Boston, Massachusetts; h Division of Translational Medicine, Eunice Kennedy Shriver National Institute of Child Health and Human Development, National Institutes of Health, Bethesda, Maryland; i Department of Pediatrics, Baylor College of Medicine, Houston, Texas; j Meyer Center for Developmental Pediatrics and Autism, Texas Children’s Hospital, Houston, Texas; k Departments of Pediatrics, Neurological Sciences, and Anatomy and Cell Biology, Rush University Medical Center, Chicago, Illinois; l Formerly Lumos Pharma, Austin, Texas; m Comprehensive Epilepsy Center, Division of Neurology, Cincinnati Children’s Hospital Medical Center, Cincinnati, Ohio; n Department of Pediatrics, University of Cincinnati College of Medicine, Cincinnati, Ohio; o Department of Radiology, Cincinnati Children’s Hospital Medical Center, Cincinnati, Ohio; p Department of Radiology, University of Cincinnati College of Medicine, Cincinnati, Ohio; q Lumos Pharma, Austin, Texas; r Department of Pediatric Behavioral Health, Primary Children’s Hospital, Salt Lake City, Utah; s Department of Pediatrics, University of Utah, Salt Lake City, Utah; t Section on Endocrinology and Genetics, National Institutes of Health, Bethesda, Maryland; u Department of Psychiatry and Behavioral Sciences, Duke University Medical Center, Durham, North Carolina; v Division of Medical Genetics, Department of Pediatrics, Duke University, Durham, North Carolina; w Division of Medical Genetics, Department of Pediatrics, University of Utah, Salt Lake City, Utah; x Department of Psychology, The Hospital for Sick Children, Toronto, Ontario, Canada; y Division of Clinical and Metabolic Genetics, Department of Pediatrics, The Hospital for Sick Children, University of Toronto, Toronto, Ontario, Canada; z Faculty of Medicine and Dentistry, Department of Medical Genetics, University of Alberta, Edmonton, Alberta, Canada; aa Department of Pediatrics, Rush University, Chicago, Illinois; ab Formerly Ultragenyx Pharmaceutical Inc., Novato, California; ac Duke Center for Autism and Brain Development, Duke University School of Medicine, Durham, North Carolina; ad Division of Developmental Medicine, Boston Children’s Hospital, Harvard Medical School, Boston, Massachusetts; ae Department of Molecular and Human Genetics, Baylor College of Medicine, Houston, Texas; af Formerly Center for Autism Research, Children’s Hospital of Philadelphia, Philadelphia, Pennsylvania; ag Division of Genetics and Genomics, Boston Children’s Hospital, Harvard Medical School, Manton Center for Orphan Disease Research, Boston, Massachusetts

**Keywords:** Creatine transporter deficiency, Intellectual disabilities, Cerebral creatine deficiency syndromes, *SLC6A8*

## Abstract

**Background:**

The purpose of the Vigilan observational study (ClinicalTrials.gov, NCT02931682) was to prospectively assess the natural history and developmental course of creatine transporter deficiency (CTD).

**Methods:**

Males with CTD aged 6 months to 65 years were evaluated at 6-month intervals for up to 4 years. Evaluations included neurodevelopmental assessments of intellectual functioning, adaptive functioning, and challenging behaviors and the onset and progression of medical comorbidities.

**Results:**

Fifty participants (median age, 7.6 years) were enrolled. The predominant CTD phenotype consisted of significant intellectual disabilities and limited skill development over time. Most participants had a history of febrile or nonfebrile seizures, gastrointestinal symptoms, and growth failure. All participants learned how to walk, 78% developed at least some verbal speech, and 34% communicated using phrases or sentences. Norm-referenced neurodevelopment assessments indicated declining standardized scores over time; however, absolute scores (i.e., age equivalent person ability scores) indicated that developmental gains were slower than average, particularly among older participants. Between-person differences in neurodevelopmental skills as a function of age did not match within-person change, suggesting a cohort effect.

**Conclusions:**

In this cohort, CTD was associated with significant and persistent intellectual disability. The use of absolute metrics from neurodevelopmental tests (e.g., person ability scores) allowed for the quantification of slow, but present, skill development.

## Introduction

Creatine transporter deficiency (CTD) is a rare X-linked creatine deficiency disorder caused by pathogenic variants in the solute carrier family 6 member 8 (*SLC6A8*) gene that encodes creatine transporter 1.^1^ Creatine transporter is expressed in a variety of tissues and is important for the uptake of creatine by cells for energy metabolism in the heart and skeletal muscles and for neuromodulation in the brain.^2–7^ It is estimated that approximately 35,000 individuals worldwide have CTD.^8^ Initial diagnosis of CTD has typically been based on elevated urinary creatine or decreased creatine signal in the brain using proton magnetic resonance spectroscopy, with confirmation by *SLC6A8* sequencing or creatine uptake assessment in fibroblasts.^5^ Initial diagnosis of CTD may be more difficult in females than in males, as urinary creatine in females may be normal or only mildly elevated.^9^ Diagnosis at early ages (i.e., before 3.5 years) typically follows observation of significant developmental delays unless there are early seizures or a known family history.^10^ Consequently, opportunities for early medical intervention will require improved early detection.^10^

CTD was first described in 2001,^1,11^ and research on the clinical phenotype has been conducted largely through retrospective or cross-sectional studies. The landmark study of 101 males with CTD by Van de Kamp and colleagues is the most comprehensive cross-sectional analysis.^12^ Males with CTD typically exhibit limited language development, limited independence with daily living skills, and intellectual disability, with a large proportion also exhibiting seizures, feeding or gastrointestinal symptoms, autism or autistic features, and challenging behaviors.^5,12,13^ There are reports of CTD in females heterozygous for CTD, and the phenotype is more variable than in males.^1,9^ The Vigilan observational study was designed to provide the first prospective, longitudinal, direct assessment of the clinical characteristics and developmental course of CTD in males. The key goals were to inform future clinical trials, including identification of developmental assessments that could serve as potential CTD outcome measures, which led to a focus on males as a more homogenous subgroup. The study complements and expands the existing cross-sectional and retrospective descriptions of the CTD phenotype in males by reporting four-year longitudinal data across selected medical, developmental, and behavioral measures.

## Materials and Methods

### Participants

Males aged 6 months to 65 years with a documented *SLC6A8* pathogenic variant were eligible. The study required traveling (pre–COVID-19) to one of nine sites in the United States and Canada. Remote data acquisition was allowed from March 2020 and beyond. Status epilepticus (seizures lasting >5 minutes) within 3 months of screening was exclusionary. Recruitment was through known clinicians serving this population and the Association of Creatine Deficiencies advocacy group.

The study (ClinicalTrials.gov, NCT02931682) was conducted in accordance with the Declaration of Helsinki and Good Clinical Practice guidelines. The institutional review board at each participating site approved the study protocol. Parents or guardians provided written informed consent for study participation.

### Study design

This observational study consisted of screening, baseline, and 48-month follow-up periods. In-person developmental and behavioral assessments were conducted every 6 months, with some semiannual visits performed remotely. Caregivers completed additional surveys every 2 months during follow-up. Screening and baseline visits could be combined. In-person assessments could be performed over 1–2 days to meet family/participant needs.

Screening data included demographics, medical history, and physical and neurological examinations. Study visits included updates to screening measures, any new medical events, a seizure tracking form, the National Institutes of Health (NIH) Gastrointestinal Questionnaire^14^ (a semistructured parent-reported history form), a neurodevelopmental assessment battery, electrocardiogram, and blood and urine sample collection. Remote check-ins via telephone or video conference every 2 months (±2 weeks) monitored changes in health status. Participants were invited to travel to the NIH (Bethesda, MD) for additional assessments (reported previously^15–18^).

This report focuses on the neurodevelopmental phenotype of the study participants, but also includes information on key clinical features (e.g., seizures and gastrointestinal symptoms), magnetic resonance imaging/magnetic resonance spectroscopy and electroencephalogram (EEG) results (when known), medication usage, and nonpharmacologic therapeutic interventions. Other histories were collected (e.g., pregnancy; birth; first year of life; health histories, including hospitalizations, surgeries, and other illnesses; feeding and sleeping histories; and family history of developmental disabilities), and a full physical examination was conducted at baseline. Some neurodevelopmental assessment battery measures were discontinued due to difficulty obtaining or interpreting data or because an alternative measure with more relevant psychometrics became available, including some biomarker assessments (e.g., wearable devices), performance-based assessments, and parent questionnaires. Some measures were piloted in preparation for future trials. The Supplemental Methods describes all neurodevelopmental measures. Supplemental Table S1 describes the disposition of each measure.

### Neurodevelopmental assessment battery

This report focuses on adaptive skills, cognitive development, and challenging behavior, which are among the most prominent developmental and behavioral features identified^12^ for which a prospective, longitudinal perspective could contribute new information.

### Adaptive skills: Vineland Adaptive Behavior Scales Interview, Third Edition

The Vineland Adaptive Behavior Scales Interview, Third Edition (Vineland-3) is a clinician-led, semistructured caregiver interview widely used to evaluate individuals with neurodevelopmental disorders and yields standard scores (population mean, 100; S.D., 15; floor, 20) for the domains of Communication, Daily Living Skills, and Socialization that are combined into an overall Vineland Adaptive Behavior Composite.^19^ Motor skills are assessed but not included in the composite score. Each domain consists of subdomains that yield scaled scores (V-scale population mean, 15; S. D., 3; floor, 1) and person ability scores (also referred to as “growth scale values” [GSVs]). Standard and scaled scores are indices of relative standing compared with participants of the same age and are not intended for quantifying change over time. GSVs are transformed raw scores intended to measure change over time in an individual and are helpful for detecting stability or subtle improvements over time in individuals with significant levels of impairment at baseline.^20,21^ Thus, norm-referenced scores are used for descriptive purposes, but quantitative analysis is based on the GSVs.

### Cognitive and developmental assessment

Given the lack of tests spanning all ages and developmental levels predicted for CTD, and consistent with other studies of heterogenous conditions with varying levels of intellectual disability,^22^ a hierarchical approach to standardized cognitive testing was used, that for the majority of the study, included the Mullen Scales of Early Learning^23^ (Mullen) and the Wechsler Abbreviated Scales of Intelligence – Second Edition^24^ (WASI-II). The Mullen is a developmental test historically used in research with neurodevelopmental conditions. It is normed for participants from birth through age 68 months and provides T-scores (mean, 50; S.D., 10; floor, 20) and mental age equivalents (MAs) in the domains of Gross Motor, Fine Motor, Visual Reception, Expressive Language, and Receptive Language. Although the WASI-II was attempted with participants aged ≥6 years (see Supplemental Material), valid scores were obtained for only four participants. Consequently, to avoid the option of no scores at all, the Mullen was administered to participants outside of the standard age range, producing MAs but not T-scores. To accommodate this, convention was followed to calculate ratio intelligence quotients (RIQ = MA/chronological age).^25^ Nonverbal mental age equivalent (NVMA) is the average of Visual Reception and Fine Motor, Verbal mental age equivalent (VMA) is the average of Expressive Language and Receptive Language, and Full Scale mental age equivalent (FSMA) is the average of NVMA and VMA. RIQs have no population distribution or floor but are colloquially interpreted on the same scale as standard scores. Use of RIQ and MA enabled quantification and evaluation of cognitive ability across the full sample, but have significant limitations.^26^

Near the end of the study, we made the decision to switch to the Bayley Scales of Infant and Toddler Development, 4^th^ Edition, and the Stanford-Binet Intelligence Scales, 5^th^ Edition, because these tests either had newer versions with updated administration features, aligned with the field’s move toward measures that include person ability scores and/or span a wide age range, have been used in neurodevelopmental disorder clinical trials, and have been accepted by FDA. We gathered sufficient data to determine these were feasible, but not enough for meaningful analysis (Bayley Scales of Infant and Toddler Development, 4^th^ Edition single-point administrations, n = 16; Stanford-Binet single-point administrations, n = 7).

### *Challenging Behavior: Aberrant Behavior Checklist* – *Second Edition*

The Aberrant Behavior Checklist – Second Edition (ABC-2) is a parent behavioral rating scale designed for individuals with intellectual disabilities and consists of five subscales: Irritability, Agitation, and Crying (15 items); Lethargy/Social Withdrawal (16 items); Stereotypic Behavior (7 items); Hyperactivity/Noncompliance (16 items); and Inappropriate Speech (4 items).^27^ Each item is scored from 0 (never a problem) to 3 (severe problem). The ABC-2 is widely used in clinical care and research and is the basis of a successful clinical indication for treatment of irritability and challenging behaviors in autism spectrum disorder.^28,29^

### Statistical analysis

R (version 4.4.2.) was used for all statistical analyses.^30^ The R packages lme4 and lmerTest were used for the generalized linear models.^31,32^ There was no formal power analysis; n = 50 was estimated as appropriate for assessing test battery utility. Medical and historical event-related data were summarized using descriptive statistics. Neurodevelopmental outcomes were depicted visually or empirically evaluated using generalized linear (growth) models, where appropriate.

In natural history studies of developmental conditions where the age at baseline—and therefore, junction of development—varies widely across participants, it is necessary to distinguish between developmental effects of age and cohort effects. A cohort effect refers to the situation in which differences between younger and older participants appear to be due to something other than the developmental course, which is especially important for rare genetic conditions in which changes in diagnostic patterns over time or ascertainment methods could induce cohort effects. Thus, neurodevelopmental data were modeled as a function of between-person age (i.e., participant’s average chronological age during the study period, centered at 8 years) and within-person time (i.e., duration of participation, 0–4 years).^33^ The between-person effect reflects differences between participants, whereas the within-person effects reflect change within a participant during the study period (referred to as “annualized change”). Uncorrelated random effects were used to account for repeated measures nested within participants (i.e., participant-level intercept and slope of within-person time) and participants nested within site.

Ten participants were siblings in four multiplex families, but the size of the dataset could not support the complexity of modeling this clustering and it was ignored. The parameters of interest from growth models were the intercept and slope estimates for the between-person and within-person effects of age, with 95% confidence intervals (CI). Plots of raw data were overlaid with individual best-fit lines to illustrate the random effects summarized by the models and to aid interpretation of the annualized change parameters and point estimates of annualized change as a function of hypothetical ages (i.e., the model-estimated annualized change for 4-, 8-, 12-, and 16-year-olds). Four participants aged >18 years were excluded from the growth models due to a lack of data coverage (Supplemental Figure S1), which restricts interpretation to participants aged ≤18 years. There was no imputation for missing data.

## Results

### Participants

Between December 12, 2016, and July 21, 2020, 50 males with CTD were enrolled (Supplemental Figure S1). The dataset was closed on March 9, 2023, and contains up to 48 months of follow-up data for each participant.

The median (interquartile range [IQR]) age at baseline was 7.6 (4.6–11.3) years, and the majority (76%) were White (Table 1). Ten participants were siblings in four multiplex families. The most common *SLC6A8* finding was deletions (n = 23). The most common first symptoms parents reported retrospectively were developmental delays before 12 months (n = 21); feeding, gastrointestinal issues, or growth failure before 12 months (n = 12); or developmental delays from 12 to 36 months (n = 8; Supplemental Table S1). Without a known family history, the median (IQR) age of first symptom in hindsight was 6.0 (3.0–12.0) months (n = 47), and the median (IQR) age of first evaluation was 15.3 (10.5–24.0) months (n = 41). The median (IQR) age of CTD diagnosis was 45.0 (24.0–84.0) months (n = 43).

Twenty-four participants saw a neurologist as one of their first specialty evaluations. EEG data were available through parent report or records on 38 participants. Referral reason was not systematically collected, but 16 of 38 EEGs were reported as normal (Table 1). At baseline, the neurological diagnoses reported by parents were hypotonia (n = 25 [50%]), including 17 diagnosed before age 2 years and 3 between ages 2–5 years; neurological weakness (n = 12 [24%]), including 9 diagnosed before age 2 years and 3 between ages 2–3 years; ataxia (n = 6 [12%]); spasticity (n = 3 [6%]); akathisia (n = 1 [2%]); dystonia (n = 1 [2%]); and tics (n = 1 [2%]).

### Seizures

**FIGURE 1.**
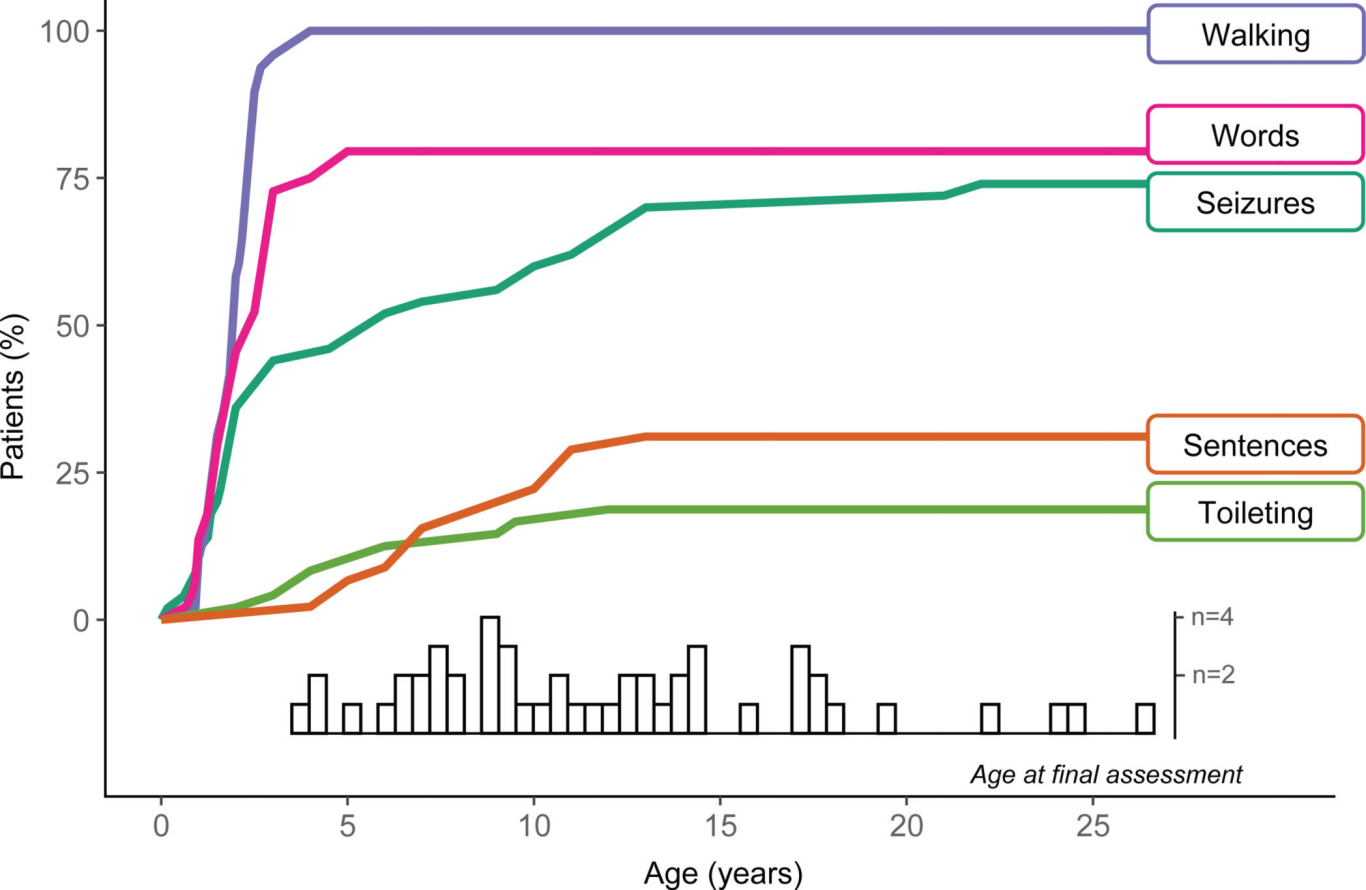
Notable milestones and events. The cumulative proportion of the sample having acquired a skill or experienced an event was plotted as a function of chronological age. The distribution of ages at final assessment is inset on the x-axis. The difference between 100% and the highest plotted percentage for a skill/event is the proportion of the sample for which the skill/event was not observed. The sample size did differ by skill/event because a participant’s data was included only if parents could recall the age of onset (i.e., if a parent reported that a participant had acquired a skill but did not recall the age, that participant was excluded from the sample for that skill). The resulting sample sizes were: walking (n = 48), first words (n = 44), first sentences (n = 45), fully toilet trained (day and night; n = 49), and onset of confirmed, nonfebrile seizures (n = 50).

Although uncontrolled seizures were an exclusion criterion, eight (16%) participants had a history of febrile seizures only, and 29 (58%) participants had a history of or current nonfebrile seizures (Table 1). In fourteen participants, nonfebrile seizures were isolated, unconfirmed, or unclear. In another 15 participants, nonfebrile seizures were more frequent and/or were being controlled with medication. Three participants had their first and only seizure (nonfebrile) during study participation, and one participant who had an isolated febrile seizure when younger developed a new seizure disorder during study participation. The median (IQR) age at first nonfebrile seizure was 3.0 (1.8–10.0) years. Figure 1 summarizes the parent-reported age of first non-febrile seizure across the sample depicted in relation to key developmental skills (i.e., language, walking, and toileting). Supplemental Table S2 notes the age of first nonfebrile seizure for each participant.

Tonic seizures were reported at baseline for 23 participants, with known onsets at age 1–3 years (n = 11), 4–7 years (n = 3), 11–13 years (n = 4), and 21–22 years (n = 2). Absence seizures were reported for 10 participants, with known onsets at age 1–2 years (n = 5), 3–6 years (n = 3), and 15 years (n = 1). Partial complex seizures were reported for six participants, with onsets at age 1–2 years (n = 3) and 6–10 years (n = 3). Some participants had more than one type of seizure.

### Imaging findings

Magnetic resonance imaging records were available for 37 participants. Among these, 25 were reported as having normal imaging, five had mild nonspecific white matter signal abnormalities, and four had thinning of corpus callosum. Three other patients had abnormal findings revealing possible focal cortical dysplasia, gyral asymmetry, and mild diffuse atrophy of cerebral hemispheres and cerebellar vermis, respectively.

Magenetic resonance spectroscopy was reported and categorizable for 25 participants. Eighteen participants had a diminished creatine peak, five had an absent or essentially absent creatine peak, and two had a normal creatine peak.

### Gastrointestinal symptoms

From the NIH Gastrointestinal Questionnaire collected at baseline and throughout study participation, 47 of 50 (94%) participants had a history of or were currently experiencing gastrointestinal symptoms. The most frequently reported symptoms were constipation (n = 41), choking/gagging (n = 38), vomiting (n = 33), growth failure (n = 33), and gastroesophageal reflux disease (n = 30; Supplemental Figure S2).

### Medication usage

Forty-eight of 50 (96%) participants received medications that were predominantly for seizure control (n = 30), gastrointestinal issues (n = 20), behavioral concerns (n = 20), or allergies (n = 16) or were CTD-relevant supplements (e.g., creatine, creatine monohydrate, glycine, arginine, and arginine hydrochloride; n = 29) or vitamins or other nutritional supplements (n = 18).

### Nonpharmacologic therapeutic interventions

Of the four broadly defined therapies (i.e., occupational, physical, speech, and behavioral therapy), 32 of 50 (64%) participants received all four, either separately or as part of a comprehensive day treatment program, nine (18%) received three therapies, six (12%) received two therapies, and two (4%) received one therapy. Other therapies included hippotherapy (n = 5) or another physical activity as therapy (e.g., aqua therapy, gymnastics, and martial arts; n = 6 each). One participant was not receiving any therapies.

### Neurodevelopmental and behavioral testing

Not all participants completed each neurodevelopmental assessment at every visit (Supplemental Figure S1B; Supplemental Table S3).

### Developmental milestones

Notable functional skills and events (i.e., walking, first words, first sentences, and toileting skills) were tracked throughout the study from all available sources, including parent reports, direct observations, and developmental testing. Figure 1 shows the cumulative proportion of participants with each skill or event by chronological age. All participants became independent walkers by age 4 years. Among the 78% of participants who could speak a word, this skill was reached by age 5 years. However, use of 2–3 word phrases or sentences was achieved by only 34% of participants, and most acquisition occurred from ages 4–13 years. Full toileting independence was achieved by 18% of participants and occurred from ages 2–9.5 years.

### Cognitive and language development

**FIGURE 2.**
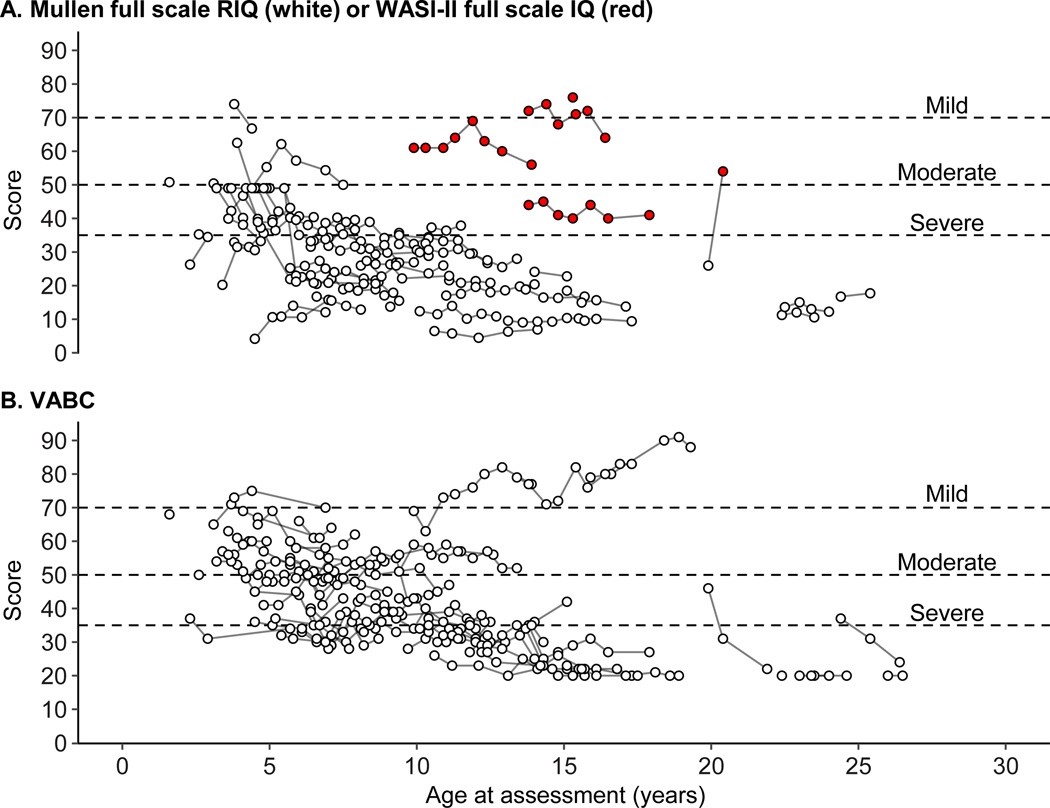
Intellectual disability measures across age. (A) Scores from the Mullen (full scale RIQ; open circles]) or WASI-II (full scale IQ [red circles]). Some participants received both tests; only the Mullen was used in statistical analyses. Because a RIQ was created with Mullen scores, the lowest possible score was zero. (B) Vineland Adaptive Behavior Composite (VABC) scores. Scores on the VABC cannot be lower than 20 (floor); thus, IQ and adaptive scores were not fully comparable. In both panels, lines connect observations within participants. Dashed horizontal lines at scores of 70, 50, and 35 indicate conventional thresholds for mild, moderate, and severe intellectual disability, respectively. IQ = intelligence quotient; RIQ = ratio intelligence quotient; WASI-II = Wechsler Abbreviated Scales of Intelligence – Second Edition; Mullen = Mullen Scales of Early Learning.

Language development could not be assessed due to an insufficient number of participants able to achieve valid scores above the normative floor of standardized testing. Full-scale Mullen and WASI-II normative scores for the assessment of cognitive development are shown in Fig 2A. Verbal and Nonverbal domain scores are shown in Supplemental Figure S3. Scores for all but two participants were in the range of intellectual disability (i.e., <70) throughout the study. The proportion of participants with severe intellectual disability (i.e., <35) increased as the sample age increased. Very few participants were able to fully complete the four-subtest WASI-II and achieve a score above the normative floor (n = 6; 12%). Among 11 participants evaluated with the Mullen and WASI-II together, the WASI-II intelligence quotient score was always higher than the Mullen RIQ score, consistent with prior observations.^34^ Thus, only the Mullen scores were used for the quantitative analysis, which excluded four participants.

**FIGURE 3.**
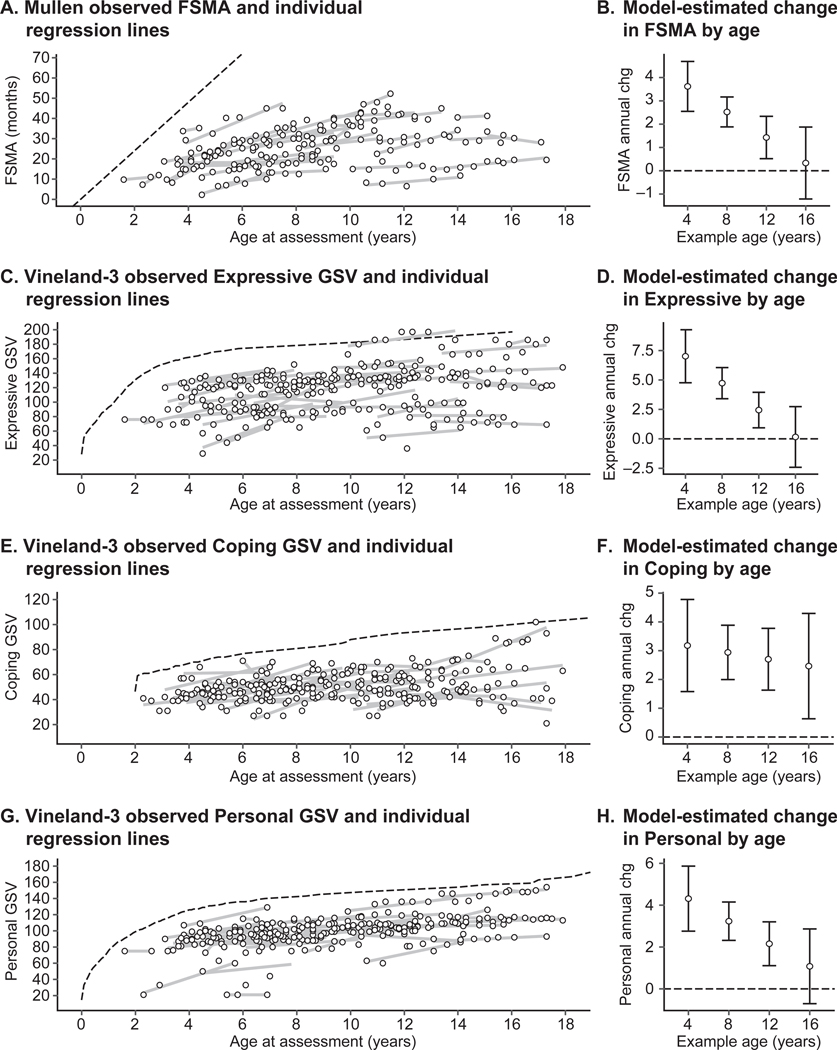
Annualized change estimates for Mullen Scales of Early Learning mental age and Vineland-3 subdomain growth scale values. Multilevel models were used to quantify the effects of between-person age and within-person time (annualized change) on the Mullen Scales of Early Learning (Mullen) mental age equivalent and the Vineland-3 GSV. Shown are the Mullen observed and model-estimated FSMA (A, B) and examples of the Vineland-3 observed and model-estimated expressive GSV) (C, D), coping GSV (E, F), and personal GSV (G, H). Observed data are shown with individual best-fit regression lines, illustrating the random portion of the multilevel model; dashed reference lines represent the age-expected score for Mullen (A) and the median GSV within the normative age group for Vineland-3 (C, E, G). Model-estimated data is shown as the estimated annualized change (chg; expected yearly change within a participant) with 95% confidence intervals at several hypothetical ages (B, D, F, H). FSMA = full-scale mental age equivalent; GSV = growth scale value; Vineland-3 = Vineland Adaptive Behavior Scales Interview, Third Edition.

Multilevel models were used to quantify annualized within-person change in Mullen FSMA, NVMA, and VMA (Fig 3A,B; Supplemental Figure S4). In all domains, the annualized rate of change within a participant was approximately 2.5 units/year and was dependent on a participant’s age, with older participants having a slower rate of change (Table 2). In all domains, the annualized within-person change was faster than the between-person differences based on age. For example, whereas the average 8-year-old was estimated to increase 2.52 (95% CI, 1.91–3.14) FSMA units per year, the difference between the average 8-year-old and the average 9-year-old was 1.16 (95% CI, 0.3–2.01) units, suggestive of a cohort effect or that differences between participants are not wholly explained by development.

### Adaptive skills

The Vineland-3 Adaptive Behavior Composite scores were almost exclusively in the range of significant impairment (i.e., <70), and the proportion of participants in the moderate and severe impairment ranges increased as the sample age increased (Fig 2B; Supplemental Figures S5 and S6). However, subdomain-level analysis of GSVs indicated some growth in all domains (see examples of Expressive, Coping, and Personal in Fig 3C-H; Supplemental Figure S7; Table 2). For Expressive Language and Personal Skills domains, annualized change was faster among younger participants than older participants and eventually stabilized. For all Vineland-3 domains, annualized change was faster than between-person differences in score, suggesting a cohort effect.

### Challenging behaviors

Although there was variability in ABC-2 subscale scores, there were significant floor effects in the Inappropriate Speech, Social Withdrawal, and Stereotypic Behavior subscales, excluding them as outcomes of interest for the general CTD population (Supplemental Figure S8). In contrast, Hyperactivity/Noncompliance and Irritability scores were relatively high, with model-estimated mean (95% CI) values of 21.39 (17.69–25.1) and 15.61 (11.62–19.60), respectively, that did not depend on age (Supplemental Table S4).

## Discussion

The Vigilan study is the first longitudinal prospective study of CTD and followed participants for up to 48 months. Natural history is critical for understanding CTD, improving clinical care, and identifying clinical outcome measures related to possible interventions.

The incidence of seizures was 74%, which is greater than that previously reported by van de Kamp and colleagues (59%),^12^ but consistent with a recent cross-sectional survey (70%).^35^ Although most nonfebrile seizures began in childhood (median, 3 years), seizures were the first identified symptom of CTD in only 6% of participants.

In two-thirds of participants, the first symptoms in hindsight were during the first year of life and included missed early milestones, growth failure, and gastrointestinal manifestations. Early, nonspecific developmental symptoms have been reported among individuals with CTD and other ultra-rare genetic conditions and have been identified as important for phenotypic descriptions and natural history studies.^10,36^

Intellectual disability was almost universally present among males with CTD in this cohort, and impairment relative to age-based peers appeared to increase with age. Despite the substantial intellectual impairment, longitudinal assessment using indicators of ability (i.e., GSV) on the Vineland rather than relative standing (i.e., norm-referenced scores) indicated that participants with CTD typically gained skills over time, consistent with clinical and family experience that individuals with CTD learn new skills, but at a much slower rate than their non-CTD peers. Given that development of skills such as phrase speech and toileting generally occurred in later childhood and that the median baseline age was 7.6 years, some participants may not have developed these skills during the study. Other studies have reported the first time development of skills such as phrase speech during adolescence.^10^

This study highlights two important methodological issues relevant to clinical CTD trial readiness. First, our results with Vineland GSV indicate that it is essential to use the appropriate score type from standardized testing to draw valid conclusions about within-person change.^21^ Second, the consistent finding of cohort effects in this sample underscores the necessity for longitudinal data in understanding the course of CTD, as cross-sectional data are likely to reflect more than the effects of development.

Consistent with extant data,^12^ parents reported a significant, but not universal, degree of irritability and hyperactivity in participants. The mean ABC-2 Irritability and Hyperactivity scores in this CTD sample are consistent with those of other genetic conditions with high rates of intellectual disability (Supplemental Table S5), and this source of commonality may be important in clinical trial planning. From a clinical perspective, these behaviors can interfere with skill development and participation in family, school, and community activities, and therefore, warrant monitoring.

All but one of the participants were actively receiving skill-based therapies or programming (e.g., speech, occupational, physical, and behavioral therapy). Mapping skill development onto a patient’s current therapy goals is beyond the scope of this study but may be an important step in understanding how medical and skills-based interventions work, either alone or in combination.

A major contribution toward clinical trial readiness for CTD was the identification of strengths and weaknesses of available clinical outcome assessments (see Supplemental Table S1). As demonstrated here and elsewhere,^12^ intellectual disability is the predominant behavioral phenotype of CTD. Qualitative work has recently identified domains most important to individuals and caregivers in CTD. The Association for Creatine Deficiencies ranked cognitive ability and adaptive functioning into their core outcome set for CTD,^37^ and a patient-focused drug development meeting demonstrated that concepts such as communicative and cognitive ability were the most highly ranked as targets for an ideal treatment.^38^ Unfortunately, relevant measures are limited, because either the age-appropriate test is too difficult or there are insufficient norms when performance is three or more S.D.s below average. The Vineland and a growing number of other standardized assessments contain person ability scores (sometimes called GSVs), which are transformations based on either Item Response Theory or Rasch analysis. Person ability scores such as the GSV have an interval-level distribution and are intended for the response monitoring context.^39^ Indeed, we were able to detect skill development when person abilities scores were available. More standardized tests are beginning to include person ability scores, which led to some changes in the protocol and suggestions for future study options (see Supplemental Table S1).

Although this study focused on the most prominent aspects of the CTD behavioral phenotype, relevant features for future investigation include characteristics of autism, detailed assessment of language among those who develop sufficient skills, and fine motor concerns. Furthermore, it may help to identify domains of relative strength among individuals with CTD, and to characterize the use of compensatory strategies such as augmentative and alternative communication devices and problem solving. Finally, we note that COVID-19 disrupted in-person visits beginning in March 2020, which impacted direct assessment data.

In conclusion, CTD is associated with significant cognitive, language, and self-help impairments that persist over time. Seizures and behavioral challenges are common but not universal. Although impairment relative to peers increases over time, incremental skill development does occur and is detectable when person ability scores or GSVs are used. Longer term studies that follow individuals with CTD into late adolescence and early adulthood are needed to discern whether the differences between age groups observed were due to limitations of assessment measures, the sample, or the course of CTD.

## Supplementary Material

MMC1

Supplementary data related to this article can be found at https://doi.org/10.1016/j.pediatrneurol.2025.10.023.

## Figures and Tables

**TABLE 1.** Demographic and Baseline Characteristics of Study Sample

Feature	N = 50

Demographics Sex, n (%) Male	50 (100)
Race/ethnicity, n (%) White	38 (76)
Asian	2 (4)
Black	2 (4)
Multiple	2 (4)
Unknown	6 (12)
Hispanic or Latino	4 (8)
Age at enrollment, years Median	7.6
Range	1.5–24.4
IQR	4.6–11.25
Age group, n (%) 1–3 years	10 (20)
4–7 years	15 (30)
8–12 years	14 (28)
13–18 years	7 (14)
19–24 years	4 (8)
Study participation Median (IQR) visits with neurodevelopmental data, n	6 (4–7)
Median (IQR) duration of study participation, years between baseline and last visit	3.55 (2.5–4)
Genetics Sequencing result, n (%) Deletion	23 (46)
Missense	11 (22)
Nonsense	4 (8)
Duplication	4 (8)
Other	3 (6)
Unknown	5 (10)
Family history of CTD, n (%)	10 (20)
Mother carrier status (44 mothers for 50 participants), n (%) Yes	12 (27)
No	15 (34)
Unknown	17 (39)
EEG results (per parent report; n = 38) Normal, n	16
Intermittent spikes in frontal and frontotemporal areas, n (%)	5
Background slowing without epileptiform activity, n (%)	3
Photo paroxysmal response, n (%)	1
Abnormal (without further description), n (%)	4
Unknown, n (%)	9
Phenotype by caregiver history Median (IQR) age at first symptom (in hindsight; n = 47), mo	6.0 (3.0–12.0)
First symptom (in hindsight; n = 50), n (%) Missed milestones or delays before 12 months	21 (42)
Feeding, gastrointestinal, or growth failure before 12 months	12 (24)
Developmental delay 12–36 months	8 (16)
Other motor atypicality	3 (6)
Seizure	3 (6)
Tested at birth because of family history	1 (2)
Visual atypicality	1 (2)
Unknown	1 (2)
Median (IQR) age at first developmental evaluation (n = 46),* mo	15.3 (6.0–27.0)
Median (IQR) age at CTD diagnosis (n = 43),* mo	48.0 (28.2–84)
Median (IQR) time since CTD diagnosis (n = 41),* mo	30.0 (19.0–70.0)
History of nonfebrile seizures, n (%)	29 (58)
Median (IQR) age of first nonfebrile seizure by history (n = 29), mo	36.0 (24.0–120)

Abbreviations:

CTD = Creatine transporter deficiency

IQR = Interquartile range

* Participants whose known family history could have been influenced this variable were excluded.

**TABLE 2.** Results of Growth Modeling of Neurodevelopmental Assessments

Outcome	Term	Expected Difference in Outcome for a One-Year Difference in Age		Expected Change in Outcome Over One Year Within a Participant	
		Age Cohort	Age Cohort ^2^	Annualized Change	Annualized Change * Age Cohort

Full scale mental age, mo	Slope (95% CI)	1.16 (0.3, 2.01)	−0.23 (−0.41, −0.04)	2.52 (1.91, 3.14)	−0.27 (−0.46, −0.09)
	Test statistic	t(41), 2.7; *P* = 0.011	t(40), −2.4; *P* = 0.019	t(30), 8.0, *P* < 0.001	t(35), −2.9; *P* = 0.006
Nonverbal mental age, mo	Slope (95% CI)	1.45 (0.58, 2.31)	−0.22 (−0.4, −0.03)	2.72 (2.04, 3.4)	−0.23 (−0.43, −0.03)
	Test statistic	t(41), 3.3; *P* = 0.002	t(40), −2.2; *P* = 0.031	t(29), 7.9, *P* < 0.001	t(34), −2.2; *P* = 0.035
Verbal mental age, mo	Slope (95% CI)	0.89 (−0.01, 1.79)	−0.25 (−0.44, −0.06)	2.33 (1.62, 3.03)	−0.31 (−0.52, −0.1)
	Test statistic	t(41), 1.9; *P* = 0.06	t(42), −2.6; *P* = 0.012	t(30), 6.5; *P* < 0.001	t(35), −2.9; *P* = 0.006
VABS expressive GSV	Slope (95% CI)	3.34 (0.26, 6.41)	−0.09 (−0.71, 0.52)	4.73 (3.46, 6.0)	−0.57 (−0.9, −0.24)
	Test statistic	t(43), 2.1; *P* = 0.039	t(43), −0.3; *P* = 0.768	t(33), 7.3, *P* < 0.001	t(34) = −3.4; *P* = 0.002
VABS coping GSV	Slope (95% CI)	0.67 (−0.49, 1.82)	0.08 (−0.15, 0.3)	2.94 (2.02, 3.86)	−0.06 (−0.3, 0.18)
	Test statistic	t(47) = 1.1; *P* = 0.263	t(43) = 0.7; *P* = 0.497	t(40) = 6.3, *P* < 0.001	t(41), −0.5; *P* = 0.627
VABS personal GSV	Slope (95% CI)	2.94 (0.97, 4.92)	0.04 (−0.35, 0.44)	3.23 (2.35, 4.12)	−0.27 (−0.5, 0.04)
	Test statistic	t(43), 2.9; *P* = 0.006	t(43), 0.2; *P* = 0.826	t(39), 7.1; *P* < 0.001	t(41), −2.3; *P* = 0.028
ABC irritability raw	Slope (95% CI)	0.7 (0.42, 1.82)	0.06 (−0.16, 0.28)	0.51 (−0.44, 1.47)	0.04 (−0.2, 0.28)
	Test statistic	t(36), 1.2; *P* = 0.229	t(35), 0.5; *P* = 0.588	t(20), 1.1; *P* = 0.304	t(21), 0.3; *P* = 0.731
ABC hyperactivity raw	Slope (95% CI)	−0.14 (−1.8, 1.51)	0.06 (−0.26, 0.38)	0.1 (−0.97, 1.17)	−0.16 (−0.43, 0.11)
	Test statistic	t(35), −0.2; *P* = 0.867	t(31), 0.4; *P* = 0.707	t(23), 0.2; *P* = 0.86	t(25), −1.1; *P* = 0.266

Abbreviations:

ABC = Aberrant Behavior Checklist

CI = Confidence interval

GSV = Growth scale value

VABS = Vineland Adaptive Behavior Scales

Table summarizes the results of multilevel models of the dependent variable (*Outcome*) as a function of age. Age is decomposed into two sources of information. The first is *Age Cohort* (between-person age), which reflects differences in the outcome between younger and older participants. Each participant has one value for this variable (the participant’s mean age during study participation), and the value was centered at 8 years. For some models, this effect was meaningfully quadratic (*Age Cohort*^*2*^ column). A negative slope for this interaction means that the degree to which a one-year difference in age was associated with a between-person difference in the outcome was diminished at older ages. The second source of information, which is the focus of interpretation, is annualized change (within-person age), which reflects changes in the outcome observed within a participant over time. Each participant had a unique value for this variable at each visit, marking the passing of time within a participant. For some models, the effect of time within a participant depended on how old the participant was, which is shown in the Annualized Change * Age Cohort column. A negative slope for that interaction means that annualized change was smaller (approaching zero) for older participants. The annualized change estimates are illustrated in Figure 3.

## Data Availability

Requests for deidentified participant data are available to researchers providing a methodologically sound proposal that is in accordance with the Ultragenyx data sharing commitment. To gain access, data requestors will need to sign a data access and user agreement. Data will be shared via secured portal.
