# Supplementary material for: Longitudinal Characterization of Males With X-Linked Creatine Transporter Deficiency: Final Results of a Multiyear Observational Study
Source: Pediatr Neurol. Author manuscript; Available in PMC 2026 Feb 14. (PMC12905711; doi:10.1016/j.pediatrneurol.2025.10.023)
Supplement: MMC1 [file NIHMS2124759-supplement-MMC1.pdf]

SUPPLEMENTAL METHODS

Performance-Based Measures of Cognition, Language, and Development

Wechsler Abbreviated Scale of Intelligence, Second Edition (WASI-II)

The WASI-II is a widely used brief test of verbal, non-verbal, and general cognitive ability for individuals aged at least 6 years.[1] The WASI-II yields standard scores (mean=100; SD=15) for a Verbal Comprehension Index, Perceptual Reasoning Index, and a Full-Scale IQ estimate. The WASI-II was attempted or ruled out based on initial observation of verbal status for all participants aged ≥6 years, with the understanding that the test would be invalid for many participants, because like most IQ tests, the WASI-II does not offer standard scores below 40, and to achieve a score that is considered valid, the test requires verbal responses.

Mullen Scales of Early Learning (Mullen)

The Mullen is a developmental test normed for children aged ≤68 months and yields an overall Early Learning Composite score and Domain scores for Gross Motor, Fine Motor, Visual Reception, Expressive Language, and Receptive Language.[2] Following common practice in neurodevelopmental disability research, the Mullen was administered to participants outside of the standard age range if they were unable to obtain a valid score on the WASI-II (described above). To accommodate assessments out of the age range and produce estimates of nonverbal and verbal abilities, convention was followed to calculate ratio intelligence quotients (RIQ = mental age / chronological age); RIQ have no population distribution or floor but are interpreted on the same scale as standard scores.[3]

Stanford Binet Intelligence Scales, Fifth Edition

The Stanford Binet Intelligence Scales, Fifth Edition, is an individually administered, norm-referenced test of intelligence and cognitive abilities for individuals aged 2−85 years and yields

an overall Full Scale IQ (FSIQ) as well as a Verbal (VIQ) and Nonverbal (NVIQ)[4]. Person ability scores (referred to as Change-Sensitive Scores) are also available.

Bayley Scales of Infant and Toddler Development, 4th Edition (Bayley-4)

The Bayley-4 is a norm-referenced developmental assessment tool used to identify developmental delays in infants and toddlers aged 16 days to 42 months and consists of five domains: Cognitive, Motor, Language, Socio-Emotional, and Adaptive Behavior, which yield norm-referenced composite scores and age equivalents.[5] Like the Mullen, the Bayley-4 is often administered outside of the standard age range if an individual is unable to obtain a valid score on a measure like the WASI-II (described above). Unlike the Mullen, person ability scores (referred to as growth scale values [GSV]) are available on the Bayley-4.

The Expressive Vocabulary Test, 2nd Edition (EVT)

The EVT–2 measures expressive vocabulary and word retrieval for individuals aged ≥2.5 years and relies on two verbal skills: the ability to say the names of objects and to generate synonyms for words.[6] EVT-2 is designed to coordinate with the Peabody Picture Vocabulary Test-4 (described below). The EVT-2 is a norm-referenced test that also provides GSV.

The Peabody Picture Vocabulary Test-4 (PPVT)

The Peabody Picture Vocabulary Test-4 is a test of receptive vocabulary for individuals aged ≥2.5 years and relies on the examinee to be able point to the picture that depicts the word the examiner says.[7] PPVT was designed to coordinate with the EVT-2, and like the EVT-2, provides norm-referenced standard scores and GSV.

NIH Toolbox – Version 2

The NIH Toolbox – Version 2 is a mostly-digital assessment of cognition, motor, sensation, and emotion for individuals aged 3−85 years.[8] For this study, we piloted a group of subtests most relevant to this population; however, they had not yet been validated in children with intellectual disabilities. Two motor subtests were included: grip strength and 9-hole pegboard dexterity test.

Eight cognitive subtests were included: Flanker Inhibitory Control Test, Dimensional Change Card Sort Test, Picture Sequence Memory, Picture Vocabulary, Oral Reading Recognition, Pattern Comparison Processing Speed, List Sorting Working Memory, and Speeded Matching. An updated Version 3 of the NIH Toolbox has been recently validated in children with ID.[9]

Parent Interview Measures of Adaptive Skills and Development

Vineland Adaptive Behavior Scales Interview, Third Edition (Vineland-3) – Comprehensive Parent Interview

The Vineland-3 is widely used to measure adaptive skills within the context of neurodevelopmental disorders and is appropriate for individuals of any age.[10] Vineland-3 yields standard scores for the domains of Communication, Daily Living Skills, and Socialization, which are combined into an overall Adaptive Behavior Composite (VABC; population mean=100; SD=15; floor, 20). Motor Skills are also assessed but are not included in the VABC. We used the Comprehensive Parent Interview form, which also yields subdomain scaled scores (V-scale population mean=15; SD=3; floor, 1) and GSV.

Developmental Profile 4 (DP-4)

The DP-4 measures development in five areas of ability: Cognitive, Social-Emotional, Adaptive Behavior, Physical, and Communication in patients from birth to an age of 21 years, 11 months.[11] The DP-4 can be administered as a Parent/Caregiver Interview Form, a Parent/Caregiver Checklist, a Teacher Checklist, or as a Clinician Rating. We used the Parent/Caregiver interview method. Norm-referenced standard scores and growth scores are available.

Parent Rating Measures of Child Behavior, Clinical Characteristics, and Parent Stress

Aberrant Behavior Checklist – Second Edition (ABC-2)

The ABC-2 is a parent rating scale designed specifically for individuals with intellectual disabilities.[12] ABC-2 yields five subscale sums: Irritability, Agitation, & Crying (15 items); Lethargy/Social Withdrawal (16 items); Stereotypic Behavior (7 items); Hyperactivity/Noncompliance (16 items); and Inappropriate Speech (4 items). Each item is scored 0 (never a problem) - 3 (severe problem) so the maximum possible varies by subscale. The ABC-2, particularly the Irritability domain, is a recognized outcome measure in clinical trials.

The Attention Deficit Hyperactivity Disorder Rating Scale-5 (ADHD-RS-5)

The ADHD-RS-5 is an 18-item rating scale used to assess DSM-5 diagnostic criteria for attention deficit hyperactivity disorder in youth aged 5−17 years. [13] ADHD-RS-5 provides separate scores for males and females, hyperactivity/impulsivity and attention, and symptoms versus impairments. We used only the parent version, but teacher versions are also available.

The Parenting Stress Index, Fourth Edition Short Form (PSI-4-SF)

The PSI-4-SF is a 36 item parent questionnaire for children aged 1 month to 12 years and measures parenting stress in three domains: child characteristics, parent characteristics, and situational/demographic life stress.[14] PSI-4-SF yields scores for Parent Distress, Parent-Child Dysfunctional Interaction, and Difficult Child, which combine to form a Total Stress score.

The Social Communication Questionnaire (SCQ)

The SCQ is a 40-item parent-reported measure of autism characteristics.[15] SCQ is based on the Autism Diagnostic Interview-Revised (ADI-R[16]) and comes in two versions: Current and Lifetime. The Lifetime version is a widely used autism screening measure focused especially on autism characteristics seen between the ages of 4 and 5 years. The Current version asks parents about characteristics seen within the last 3 months. Although not as widely used as a screener, we used the Current version of the SCQ in this study because of the significant early

delays experienced by most patients with CTD, and because of the general difficulty distinguishing significant intellectual disabilities from autism at younger ages.

Clinician Ratings

Clinical Global Impression – Severity (CGI-S) and Clinical Global Impression – Improvement (CGI-I)[17,18]

CGI-S is used to rate a person's illness on a 7-point scale from 1=not at all ill, to 7=among the most extremely ill. CGI-I is used to rate the extent to which the illness has improved or worsened relative to Baseline on a 7-point scale from very much improved (1) to very much worse (7). In a longitudinal study, CGI-S is rated independently at baseline and each study visit, whereas CGI-I is rated at each study visit in relation to the original baseline CGI-S. CGI's can be adapted to target specific aspects of an illness or the total clinical presentation. For this study, CGI-S was based on the following question: "Considering your total clinical experience with children and adults (with and without developmental disorders), how severe are the participant's behavioral, emotional, or attentional challenges (regardless of development level)." Information from all sources could be included: direct observation, medical history, caregiver report of home, school, and community functioning and information gathered through study assessments and performance-based measures. At each study visit, an updated CGI-S score was rated and a CGI-Improvement scale was rated to assess how much the participant's behavioral, emotional, or attentional challenges had changed relative to baseline.

Developmental Disabilities Children's Global Assessment Scale (DD-CGAS)

The DD-CGAS was modified from the Children's Global Assessment Scale (CGAS[20]).[19] DD-CGAS is a clinician-rated scale yielding a single score from 1–100 that indicates the global functioning of a youth with a developmental disability relative to youth of the same age but without a developmental disability. Four domains are considered in the clinician's overall score:

Self-Care, Communication, Social Behavior and School/academic functioning. Each 10-point increment on the scale from 1–100 includes descriptions of the skills and level of support generally needed within that score range.

SUPPLEMENTAL FIGURES

Figure S1. Participant dispensation and data coverage. A, Participant dispensation through study visits. Four participants discontinued early for personal reasons. Otherwise, the study stopped data collection after 6 years of data collection but before all participants had reached the 48-month visit. Some visits were also missed due to pandemic-related shutdowns. **B**, Neurodevelopmental data coverage. Each dot indicates that at least one neurodevelopmental assessment (Vineland, Mullen, WASI, and/or ABC) was available for the participant at a given study interval, indicated by color. Visits per participant are connected with a horizontal line and participants are arranged by age at baseline.

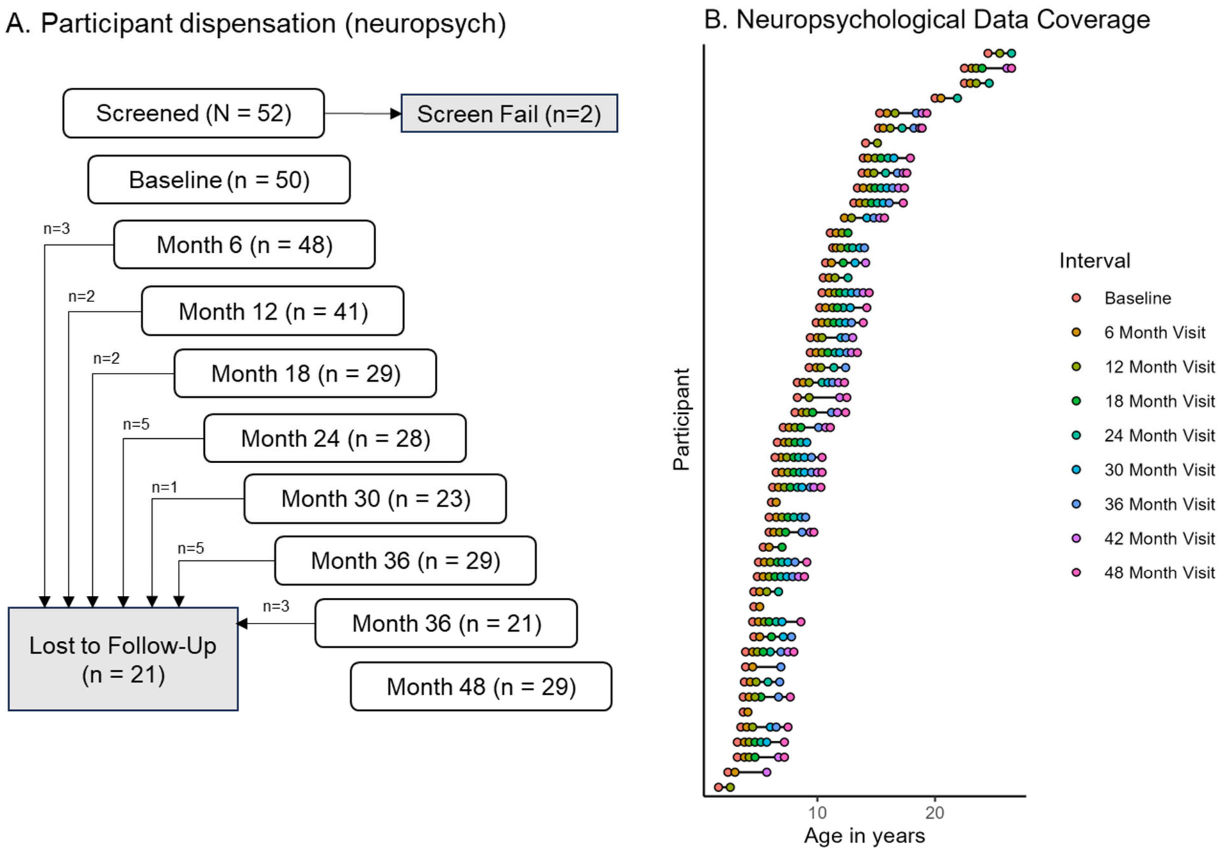

Figure S2. NIH Gastrointestinal Questionnaire. Caregiver responses from baseline and at any study visits were combined to determine whether the participant had or had not ever experienced this symptom. Items related to incontinence were excluded because many individuals with CTD do not achieve independent toileting.

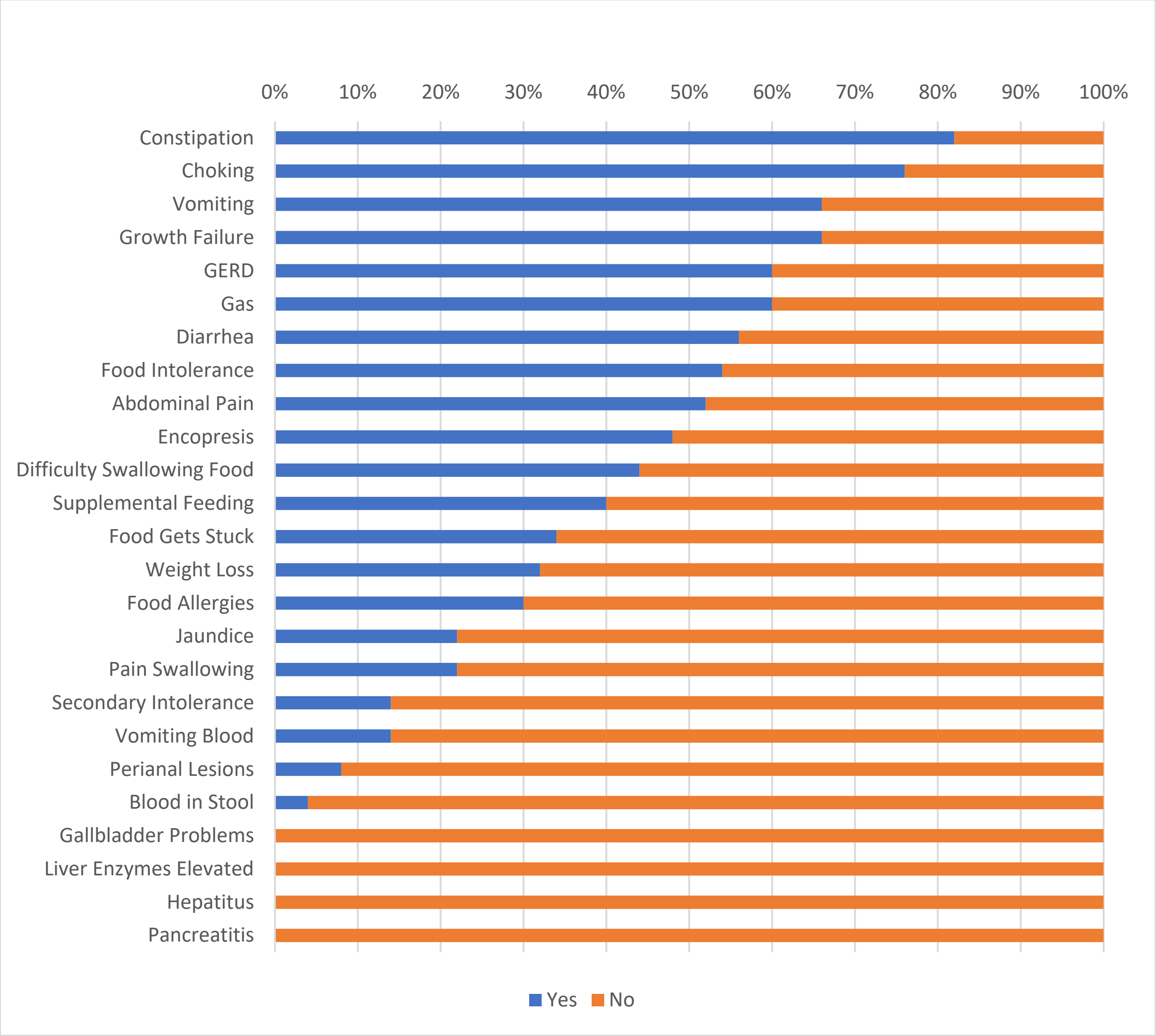

Figure S3. IQ (WASI-II) and RIQ (MSEL) overall and domain scores. Observations within participants are connected by a solid gray line. IQ scores have a population mean of 100 and SD of 15. RIQ do not have population-level distributions but are interpreted on the same scale of borderline (70–85), mild (50–70), moderate (35–50), and severe/profound (<35), indicated by the gray dotted lines.

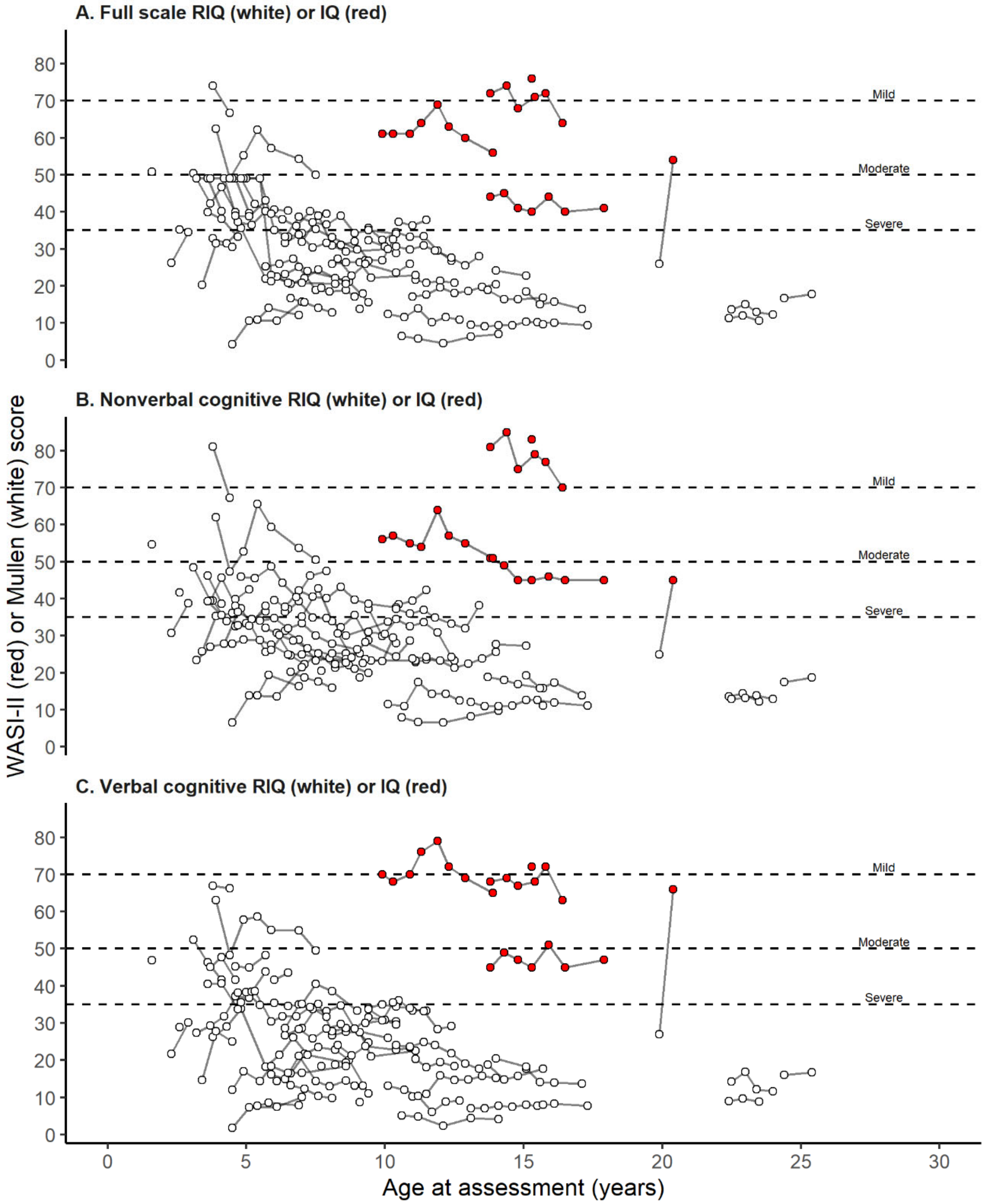

Figure S4. Vineland Domain Standard Scores. Observations within participants are connected by a solid gray line. To aid comparability to the IQ data, the participants who received a WASI in Figure S3 are also indicated here with red circles. Normative data are represented by a dotted red line. The population average of standard scores is 100 (SD=15).

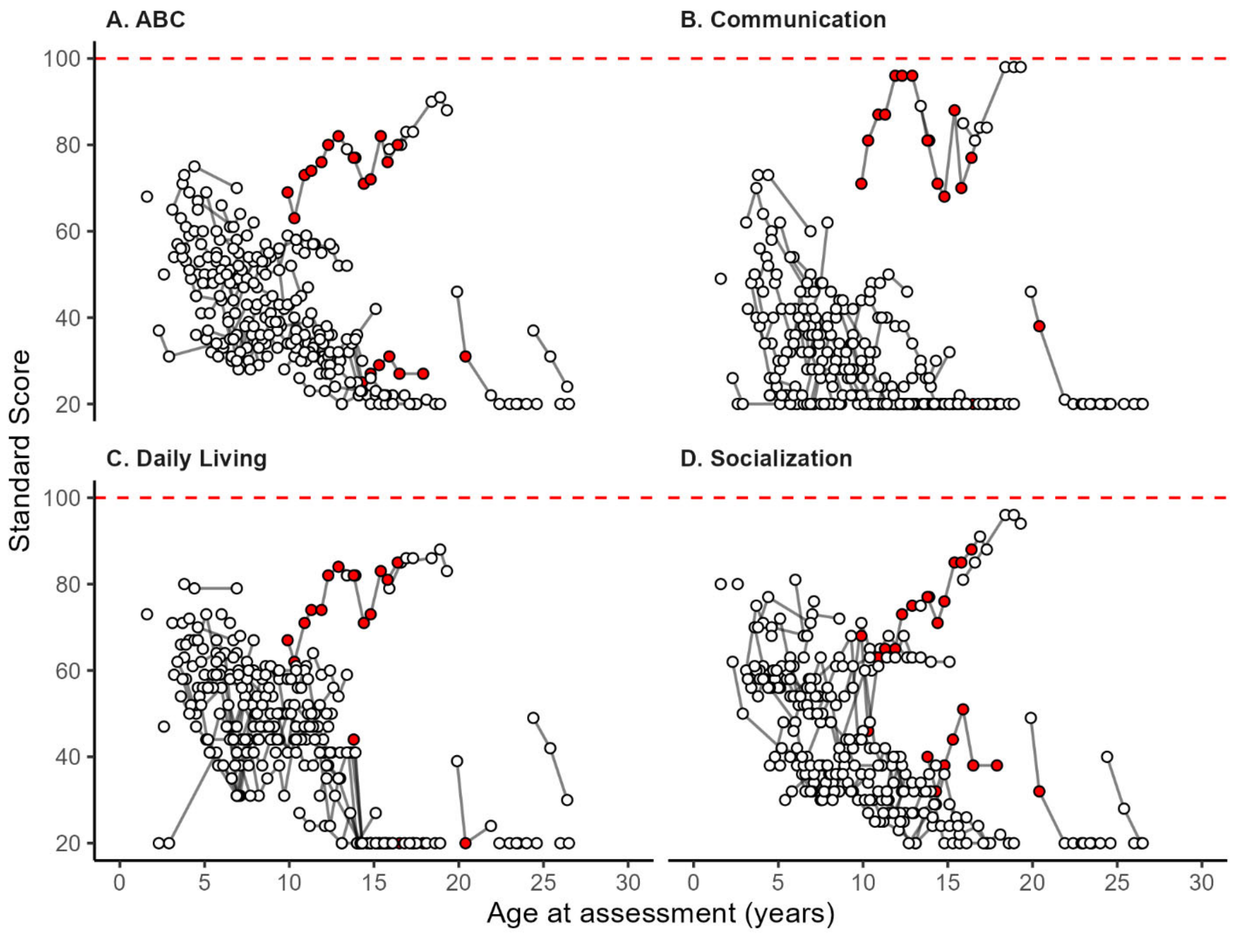

Figure S5. Vineland subdomain V-scale scores. Observations within participants are connected by a solid gray line. To aid comparability to the IQ data, the participants who received a WASI in Figure S3 are also indicated here with red circles. Normative data are shown by a dotted red line. The population average of V-scale scores is 15 (SD=3).

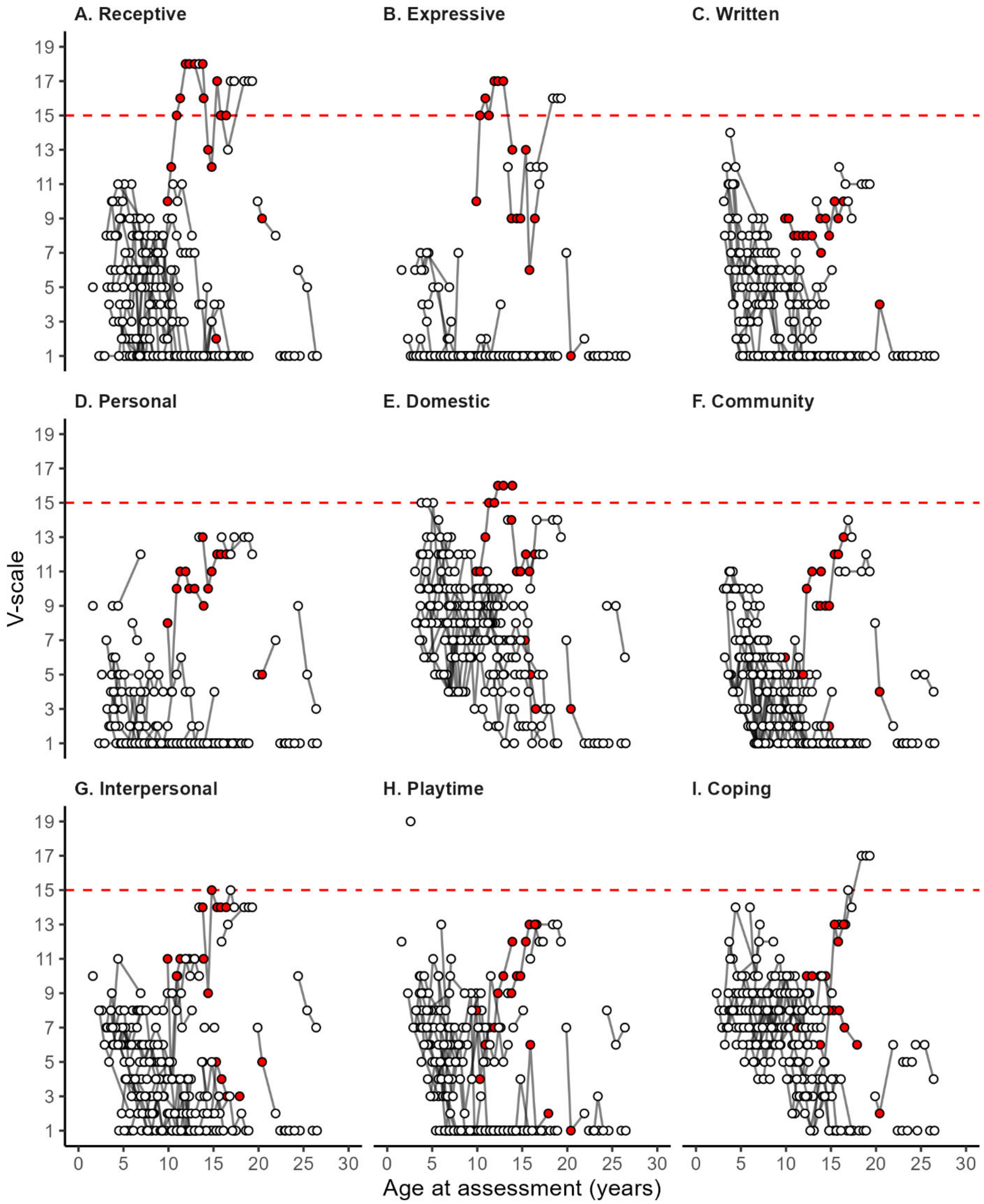

Figure S6. Vineland subdomain growth scale values (GSV). Observations within participants are connected by a solid gray line. Normative data are shown by dotted red line: the median GSV value per available age equivalent. GSVs are not comparable across subdomains.

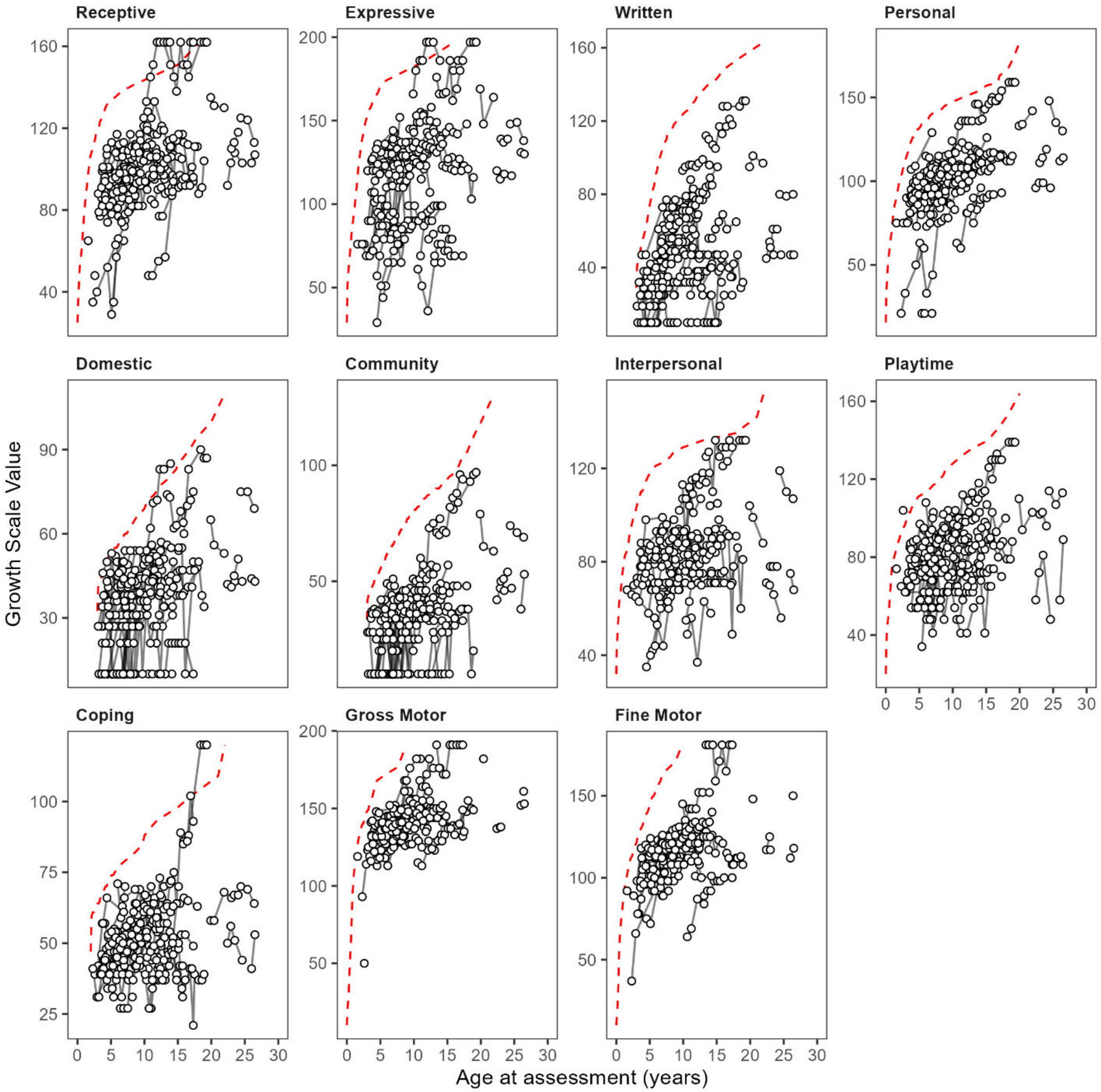

Figure S7. Aberrant Behavior Checklist (ABC-2) raw subscale scores. ABC-2 scores within participants (connected by solid lines) are shown as a function of age.

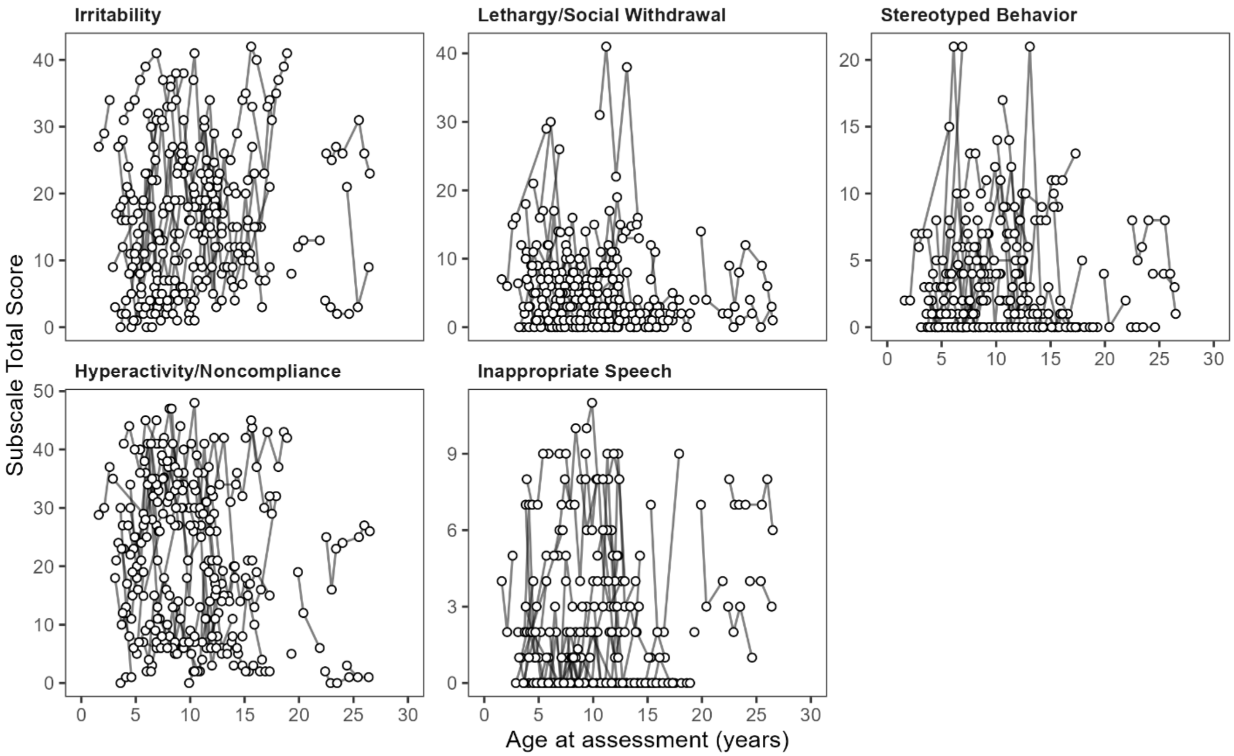

SUPPLEMENTAL TABLES

Supplemental Table S1. Measures used or piloted in Vigilan.

Name	Area	Disposition	Reason
**Performance-Based Measures**			
**Bayley**	Cognition and development	Piloted at end of study (n=16 single-participant administrations); seems promising as option in hierarchy of tests	Person ability scores available; can be administered out of age range
**EVT**	Expressive language	Piloted at end of study (n=21 participants could receive person ability scores); potentially useful	Person ability scores available; co-normed with PPVT
**Mullen**	Cognition and development	Used in current report but would be replaced with Bayley in future studies	No person ability score available; measure not updated
**NIH Toolbox-4**	Cognition	Piloted but discontinued	Version 4 tasks were too difficult for CTD; Version 5 is being validated in intellectual disability
**PPVT**	Receptive language	Piloted at end of study (n=32 participants could receive a person ability score); potentially useful	Person ability scores available, co-normed with EVT
**Stanford-Binet-5**	Cognition	Piloted at end of study (n=7), seems promising as option in hierarchy of tests	Person ability scores available, test spans a wide age range, has z-deviation scores for VIQ, NVIQ and FSIQ to avoid floor effects
**WASI-II**	Cognition	Used in current report but would be replaced with Stanford-Binet-5 in future studies	Too difficult for most CTD patients; no person ability scores
**Parent Interviews**			
**DP-4**	Development	Piloted (n=10 with 2–3 administrations); seems promising	Person ability scores available

**Vineland-III**	Adaptive skills	Used in current report and would continue to use in future studies	Person ability scores available
**Parent Questionnaires**			
**ABC-2**	Interfering behaviors	Used in current report and would continue	Relevant behaviors measured in context of intellectual disability
**ADHD-RS-5**	Attention, impulsivity, and hyperactivity	Discontinued	Items were difficult for parents to rate in the context of intellectual disability
**PSI-4 (Short Form)**	Parent stress	Discontinued	Items were difficult for parents to rate in the context of intellectual disability
**SCQ (Current)**	Autism characteristics	Piloted; could be part of a full autism assessment	Autism and significant intellectual disability are difficult to differentiate without a more complete assessment
**Clinician Ratings**			
**CGI-S & CGI-I**	Current level of impairment and improvement since baseline	Piloted; shows promise with caveats	Further reliability exercises needed
**DD-CGAS**	General level of functioning	Piloted; shows promise with caveats	Further reliability exercises needed
**Wearable Biomarker Assessments**			
**Echocardiogram**		Results reported elsewhere[21]	Patient tolerance was acceptable
**Seizure Monitoring**	Embrace2 wrist-worn wearable	Discontinued	Patient tolerance was low in our sample
**Event-related potentials**	Cognision®	Discontinued	Patient tolerance was low to moderate; child comparison data is not yet available

Other Measures

If families chose to also visit the NIH, they received an MRS, lumbar puncture for cerebrospinal fluid collection, overnight EEG, and skin biopsies, which were all performed under sedation. Results from these extra procedures conducted at the NIH are reported elsewhere.[21–23]

Supplemental Table S2. Participant-level first symptoms (in hindsight) and ages of evaluation and diagnosis

		Age, years			
Participant	First Symptom	First Symptom	First Evaluation	CTD Diagnosis	First Non-Febrile Seizure
1	Speech delay	1.5	2.5	4.0	3.0
2	Motor delay	0.75	3	3.0	n/a
3	Feeding/gastrointestinal	0.003	2	4.0	n/a
4	Abnormal muscle development	U	2.25	4.5	1.5
5	Speech delay	2.5	2.5*	5.0*	n/a
6	Motor delay	0.75	1.25	1.6	1.25
7	Motor delay	0.75	0.75	3.0	n/a
8	Motor delay	0.5	2	2.0	2.0
9	Motor delay	0.5	0.5	5.5	4.5
10	Hypotonia	0.33	8	8.0	2.0
11	Motor delay	1.0	1.3	3.75	n/a
12	Motor delay	1.5	5	10.0	10
13	Motor delay	0.5	1	3.0	5
14	Abnormal posturing	0.17	0.17	3.4	3
15	Failure to thrive	1	5*	6.0*	10.0
16	Failure to thrive	0.33	U	1.42	3.0
17	Tested at birth	n/a*	0.17*	0.17*	n/a
18	Suspected visual impairment	0.08	3	13.0	13.0
19	Failure to thrive, feeding/gastrointestinal	0.03	1.4	2.5	7
20	Motor delay	0.33	0.75	2.2	n/a
21	Failure to thrive, feeding/gastrointestinal	0	1	10.0	22.0
22	Feeding/gastrointestinal	0.08	1.25	2.5	3.0
23	Feeding/gastrointestinal	0.03	0.4	6.0	6.0
24	Motor delay	0.25	0.75	6.0	n/a
25	Motor delay	0.5	1.5	4.0	n/a
26	Motor delay	0.25	0.5	2.0	n/a
27	Speech delay	1.5	4	6.0	n/a
28	U	U	U	6.0	n/a
29	Motor delay	0.5	0.5	0.7	9.0
30	Motor delay	0.5	2	7.0	n/a
31	Failure to thrive	0.25	1	2.0	1.58
32	Seizure	1.0	1	1.0	1.0

33	Feeding/gastrointestinal	0.02	0.05	13.0	13.0
34	Motor delay	1.0	2	7.0	6.0
35	Speech delay	<3	U	U	n/a
36	Motor delay	1.3	6*	7.0*	n/a
37	Seizure	2.0	2	7.0	n/a
38	Motor delay	0.75	2.5	6.0	21.0
39	Motor delay	0.5	1	9.0	n/a
40	Global developmental delay	0.9	1	2.5	n/a
41	Motor delay	0.75	2	2.0	n/a
42	Motor delay	1.0	<2	8.0	12.0
43	Motor and speech delay	1.0	1	2.75	0.6
44	Developmental and speech delay	1.0	1	17.0	1.0
45	Speech delay	2.0	3	3.0	n/a
46	Failure to thrive	0.3	0.5	0.9	2
47	Feeding/gastrointestinal	0.03	0.03*	0.25*	n/a
48	Feeding/gastrointestinal, exaggerated startled response	0.03	0.03*	0.03*	2.0
49	Global developmental delay	1.3	1.3	1.5	12.0
50	Seizure	0.01	1	1.0	0.17

CTD, creatine transporter deficiency; U, unknown.

*First evaluation and age of diagnosis may have been influenced by having a known family history of CTD. Note that in some situations, the oldest patient in a multiplex family was not the first to be diagnosed, and that genetic testing was sometimes not conducted on infant siblings until there was a developmental concern.

Supplemental Table S3. Summary of data available for each neurodevelopmental measure.

Measure	Participants with at least one assessment, n	Median (IQR) assessments per person, n	Median (IQR) age at first assessment, years	Median (IQR) time between first and last assessment, years
Vineland Adaptive Behavior Scale	50	6.0 (4.0-7.0)	7.6 (4.6-11.3)	3.6 (2.5-4.0)
Mullen Scales of Early Learning	46	4.0 (3.0-6.8)	6.5 (4.5-10.6)	2.1 (1.0-3.0)
Weschler Abbreviated Scale of Intelligence	16	2.0 (2.0-4.5)	13.8 (9.4-15.2)	1.1 (0.6-2.2)
Aberrant Behavior Checklist	50	5.0(4.0-7.8)	7.6 (4.7-11.1)	3.8 (2.1-4)

Some participants received both the Mullen Scales of Early Learning and the Weschler Abbreviated Scale of Intelligence at the same visit. For quantitative analysis, the Mullen was used, if available.

Supplemental Table S5. Aberrant Behavior Checklist subscale scores in other genetic conditions associated with intellectual disabilities.

Citation	Genetic Condition	n	Mean (SD) Age, years	Age Range, years	Intellectual Disability	Mean (SD) Irritability	Mean (SD) Lethargy / Social Withdrawal	Mean (SD) Inappropriate Speech	Mean (SD) Hyperactivity	Mean (SD) Stereotypic Behavior
Clarke & Marston (2000)[24]	Angelman Syndrome	73	11 (5.7)	5 to 33	78% in severe range	8.74 (7.64)	4.93 (5.27)	0.43 (1.11)	20.08 (11.38)	4.35 (4.45)
Summers & Feldman (1999)[25]	Angelman Syndrome	27	9.08 (5.75)	2 to 25	74% in severe or profound range	4.48 (5.34)	4.59 (4.53)	0.37 (0.38)	14.41 (9.49)	3.00 (3.66)
Oliver et al. (2008)[26]	Cornelia de Lange	54	13.88 (8.58)	3 to 38	76% severe or profound	10.63 (9.37)	9.12 (10.07)	1.59 (2.77)	13.02 (12.68)	4.23 (5.27)
Clarke & Boer (1998)[27]	cri du chat	38	17.3	5 to 40	85% in severe range (reported by caregivers)	13.21 (9.05)	5.24 (6.85)	2.39 (3.48)	21.68 (10.88)	5.29 (5.25)
Dykens & Clarke (1997)[28]	cri du chat	146	12 (8.93)	2 to 40	"Mild to profound delay"	11.05 (8.13)	4.18 (6.14)	1.46 (2.25)	17.86 (10.69)	4.66 (4.53)
Capone et al. (2008)[29]	Down Syndrome	23	7.8 (2.6)	3 to 13	"Moderate to profound cognitive impairment"	15.8 (10.7)	22.0 (9.8)	2.7 (3.4)	26.4 (10.7)	12.5 (4.9)
Capone et al. (2016)[30]	Down Syndrome	23	7.4 (4.1)	4 to 12	Mild to severe	13.0 (6.7)	3.6 (3.1)	1.5 (1.7)	29.9 (7.9)	2.5 (2.5)
Stores et al. (1998)[31]	Down Syndrome	91	10.5	4 to 19	Not reported	5.56	3.96	2.16	9.86	1.75
Gothelf et al. (2008)[32]	Fragile X	84	11.7 (5.7)	1 to 22	Mean, 69.7; SD, 20.8;	5.50 (6.70)	3.20 (4.10)	2.1 (2.2)	7.7 (7.6)	1.6 (2.6)

					range, 40−116					
Kau et al. (2000)[33]	Fragile X	41	Not reported	3 to 6	Mean, 56.41; SD, 14.18	9.34 (6.54)	3.87 (3.66)	Not reported	17.89 (8.50)	Not reported
Clarke & Boer (1998)[27]	Prader Willi	55	21.1	6 to 43.5	48% in moderate range; 42% in mild	14.59 (11.90)	6.71 (7.59)	4.20 (3.44)	7.60 (6.27)	1.55 (2.49)
Clarke & Boer (1998)[27]	Smith Magenis	21	14.5	5 to 33	63% in moderate range	21.67 (11.16)	7.47 (5.29)	6.10 (3.66)	26.10 (10.13)	7.00 (4.55)
Vigilan (baseline)	Creatine Transporter Deficiency	50	8.8 (5.4)	1.6 to 24	Mean, 32.2; SD, 17.0	14.99 (10.83)	6.70 (6.53)	2.28 (3.01)	21.11 (14.19)	3.52 (4.01)

Intellectual disabilities are neurodevelopmental disorders that begin in childhood and are characterized by intellectual difficulties and difficulties in conceptual, social, and practical areas of living.[34]

REFERENCES

1. Wechsler D. *Wechsler Abbreviated Scale of Intelligence, Second Edition*. NCS Pearson; 2011.

2. Mullen EM. *Mullen Scales of Early Learning*. American Guidance Service, Inc; 1995.

3. Thurm A, Tierney E, Farmer C, et al. Development, behavior, and biomarker characterization of Smith-Lemli-Opitz syndrome: an update. *J Neurodev Disord*. 2016;8(1):12. doi:10.1186/s11689-016-9145-x

4. Roid GH. *Stanford-Binet Intelligence Scales, Fifth Edition.* Riverside Publishing; 2003.

5. Bayley N, Aylward GP. *Bayley Scales of Infant and Toddler Development, Fourth Edition.* NCS Pearson

6. Williams KT. *Expressive Vocabulary Test Second Edition*. Pearson; 2007.

7. Dunn LM, Dunn DM. *Peabody Picture Vocabulary Test, Fourth Edition*. NCS Pearson; 2007.

8. Gershon RC, Wagster MV, Hendrie HC, Fox NA, Cook KF, Nowinski CJ. NIH Toolbox for Assessment of Neurological and Behavioral Function. *Neurology*. 2013;80(11 Suppl 3):S2-S6. doi:10.1212/WNL.0b013e3182872e5f

9. Shields RH, Kaat AJ, McKenzie FJ, et al. Validation of the NIH Toolbox Cognitive Battery in intellectual disability. *Neurology*. 2020;94(12):e1229-e1240. doi:10.1212/WNL.0000000000009131

10. Sparrow SS, Cicchetti DV, Saulnier, CA. *Vineland Adaptive Behavior Scales, Third Edition.* American Guidance Service, Inc; 2016.

11. Alpern GD. *Developmental Profile 4 (DP-4).* Western Psychological Services; 2020.

12. Aman MG, Singh NN. *Aberrant Behavior Checklist Second Edition*. Slosson Educational Publications, Inc.; 2017.

13. DuPaul GJ, Power TJ, Anastopoulos AD, Reid, Robert. *ADHD Rating Scale-5 for Children and Adolescents: Checklists, Norms, and Clinical Inpterpretation*. Guilford Publications; 2016.

14. Abidin RR. *Parenting Stress Index, Fourth Edition Short Form*. PAR, Inc; 2016.

15. Rutter M, Bailey A, Lord, Catherine. *The Social Communication Questionnaire*. Western Psychological Services; 1994.

16. Rutter M, LeCouteur A, Lord, Catherine. *Autism Diagnostic Interview-Revised (ADI-R)*. Western Psychological Services; 2003.

17. Guy W. Clinical Global Impressions. In: *ECDEU Assessment Manual for Psychopharmacology, Revised*. US Department of Health, Education, and Welfare; 1976:217-222.

18. Busner J, Targum, S. The Clinical Global Impressions Scale: Applying a Research Tool in Clinical Practice. *Psychiatry*. Published online July 2007:28-37.

19. Wagner A, Lecavalier L, Arnold LE, et al. Developmental disabilities modification of the Children's Global Assessment Scale. *Biol Psychiatry*. 2007;61(4):504-511. doi:10.1016/j.biopsych.2007.01.001

20. Shaffer D, Gould MS, Brasic J, et al. A Children's Global Assessment Scale (CGAS). *Arch Gen Psychiatry*. 1983;40(11):1228-1231. doi:10.1001/archpsyc.1983.01790100074010

21. Levin MD, Bianconi S, Smith A, et al. X-linked creatine transporter deficiency results in prolonged QTc and increased sudden death risk in humans and disease model. *Genet Med*. 2021;23(10):1864-1872. doi:10.1038/s41436-021-01224-8

22. Rahhal S, Farmer C, Thurm A, et al. Corrigendum to "Elevated amyloid beta peptides and total tau in cerebrospinal fluid in individuals with Creatine transporter deficiency." *Mol Genet Metab Rep*. 2023;37:101013. doi:10.1016/j.ymgmr.2023.101013

23. Abdennadher M, Inati SK, Rahhal S, et al. Characterization of seizures and EEG findings in creatine transporter deficiency due to SLC6A8 mutation. *Am J Med Genet A*. 2024;194(2):337-345. doi:10.1002/ajmg.a.63418

24. Clarke DJ, Marston G. Problem behaviors associated with 15q- Angelman syndrome. *Am J Ment Retard AJMR*. 2000;105(1):25-31. doi:10.1352/0895-8017(2000)105<0025:PBAWQA>2.0.CO;2

25. Summers JA, Feldman MA. Distinctive pattern of behavioral functioning in Angelman syndrome. *Am J Ment Retard AJMR*. 1999;104(4):376-384. doi:10.1352/0895-8017(1999)104<0376:DPOBFI>2.0.CO;2

26. Oliver C, Arron K, Sloneem J, Hall S. Behavioural phenotype of Cornelia de Lange syndrome: case-control study. *Br J Psychiatry J Ment Sci*. 2008;193(6):466-470. doi:10.1192/bjp.bp.107.044370

27. Clarke DJ, Boer H. Problem behaviors associated with deletion Prader-Willi, Smith-Magenis, and cri du chat syndromes. *Am J Ment Retard AJMR*. 1998;103(3):264-271. doi:10.1352/0895-8017(1998)103<0264:PBAWDP>2.0.CO;2

28. Dykens EM, Clarke DJ. Correlates of maladaptive behavior in individuals with 5p- (cri du chat) syndrome. *Dev Med Child Neurol*. 1997;39(11):752-756. doi:10.1111/j.1469-8749.1997.tb07377.x

29. Capone GT, Goyal P, Grados M, Smith B, Kammann H. Risperidone use in children with Down syndrome, severe intellectual disability, and comorbid autistic spectrum disorders: a naturalistic study. *J Dev Behav Pediatr JDBP*. 2008;29(2):106-116. doi:10.1097/DBP.0b013e318165c100

30. Capone GT, Brecher L, Bay M. Guanfacine Use in Children With Down Syndrome and Comorbid Attention-Deficit Hyperactivity Disorder (ADHD) With Disruptive Behaviors. *J Child Neurol*. 2016;31(8):957-964. doi:10.1177/0883073816634854

31. Stores R, Stores G, Fellows B, Buckley S. Daytime behaviour problems and maternal stress in children with Down's syndrome, their siblings, and non-intellectually disabled and other intellectually disabled peers. *J Intellect Disabil Res JIDR*. 1998;42 (Pt 3):228-237. doi:10.1046/j.1365-2788.1998.00123.x

32. Gothelf D, Furfaro JA, Hoeft F, et al. Neuroanatomy of fragile X syndrome is associated with aberrant behavior and the fragile X mental retardation protein (FMRP). *Ann Neurol*. 2008;63(1):40-51. doi:10.1002/ana.21243

33. Kau AS, Reider EE, Payne L, Meyer WA, Freund L. Early behavior signs of psychiatric phenotypes in fragile X syndrome. *Am J Ment Retard AJMR*. 2000;105(4):286-299. doi:10.1352/0895-8017(2000)105<0286:EBSOPP>2.0.CO;2

34. American Psychiatric Association. *Diagnostic and Statistical Manual of Mental Disorders - Fifth Edition, Text Revision - DSM-5-TR*. 5th, text revision ed. American Psychiatric Association Publishing; 2022.
